# Les pyélonephrites aigues de la femme enceinte: place du traitement médical et indications d'un drainage de la voie excrétrice supérieure (y'a-t-il des facteurs prédictifs cliniques, biologiques et radiologiques pour rendre le drainage licite?)

**DOI:** 10.11604/pamj.2015.22.324.7262

**Published:** 2015-12-02

**Authors:** Abdessamad El Bahri, Abdellatif Janane, Jaouad Chafiki, Tayiri Arnaud, Mohammed Ghadouane, Ahmed Ameur, Mohammed Abbar

**Affiliations:** 1 Service d'Urologie, Hôpital Militaire d'Instruction Mohammed V, Rabat, Maroc

**Keywords:** Pyélonéphrite aigue, gestation, sonde double, Acute pyelonephritis, gestation, double probe

## Abstract

Les pyélonéphrites aigues gravidiques (PNAg) sont fréquentes et peuvent avoir des conséquences maternelles et fœtales graves. Le but de notre étude était de déterminer les facteurs prédictifs cliniques, biologiques et radiologiques qui permettent de se limiter au traitement médical ou d'associer un drainage de la voie excrétrice supérieure dans la prise en charge des PNAg. Nous rapportons, de façon rétrospective une série de 26 cas dans les services d'Urologie et de Gynécologie de l'Hôpital Militaire d'Instruction Mohamed V de Rabat (Maroc) sur une période allant du 1er Janvier 2010 au 30 Aout 2012. Toutes les patientes avaient une PNAg symptomatique objectivée par l'ECBU et/ou l’échographie rénale. La fréquence de la pyélonéphrite aigue gravidique par rapport aux pyélonéphrites aigues en général a été de 27,95% avec une prédominance chez les primipares de 53,84%. Son pic de fréquence se situe à 73,08% pour les gestantes âgées de 19 à 37 ans ainsi qu'au troisième trimestre (77%) de la grossesse. La triade clinique fièvre, lombalgie, troubles mictionnels et l’échographie rénale sont les éléments importants du diagnostic. L'antibiothérapie probabiliste a été débuté d'emblée et adaptée en fonctions des résultats de l'examen cytobactériologique des urines. Sa durée est de trois à six semaines en fonction de l’évolution clinique. La protéine C réactive est un marqueur de progression de la maladie ou de l'efficacité thérapeutique. Les principaux facteurs prédictifs du drainage de la voie excrétrice supérieure sont: persistance de la symptomatologie clinique, du syndrome infectieux et des anomalies visibles à l’échographie rénale ainsi que l'altération de la fonction rénale. La montée de la sonde JJ est le principal traitement urologique. Le traitement médical repose sur l'antibiothérapie probabiliste qui sera adaptée Ultérieurement en fonction des résultats de l'antibiogramme. Les facteurs prédictifs d'un drainage de la VES sont: la persistance de la symptomatologie clinique, du syndrome infectieux et des anomalies visibles à l’échographie rénale ainsi que l'altération de la fonction rénale.

## Introduction

Les pyélonéphrites aigues gravidiques sont fréquentes et peuvent avoir des conséquences maternelles et fœtales graves. Leur diagnostic est évoqué devant la triade fièvre, lombalgies, troubles mictionnels. Le germe le plus fréquemment retrouvé est l'Escherichia coli. La pyélonéphrite aigue gravidique est une urgence médico-chirurgicale compte tenu de ces conséquences potentielles. Le but de notre article était de déterminer les facteurs prédictifs cliniques, biologiques et radiologiques qui permettent de se limiter au traitement médical ou d'associer un drainage de la voie excrétrice supérieure dans la prise en charge des pyélonéphrites aigues gravidiques.

## Méthodes

Nous avons mené une étude rétrospective de la pyélonéphrite aigue au cours de la grossesse à propos de 26 cas dans les services d'Urologie et de Gynécologie de l'Hôpital Militaire d'Instruction Mohamed V de Rabat. Elle s'est étalée sur une période allant du 1er Janvier 2010 au 30 Aout 2012. Ont été inclus dans notre étude toutes les patientes avec PNAg symptomatique objectivée par l'ECBU et/ou l’échographie rénale. Les patientes jeunes en âge de procréation et toutes les patientes de la cohorte répondaient aux paramètres de la fiche d'exploitation. Ont été exclues de notre étude les patientes avec cystite isolée, apyrétique avec ECBU de contrôle non documenté et les infections nosocomiales à l'origine de la PNA gravidique.

## Résultats

**Figure 1:**
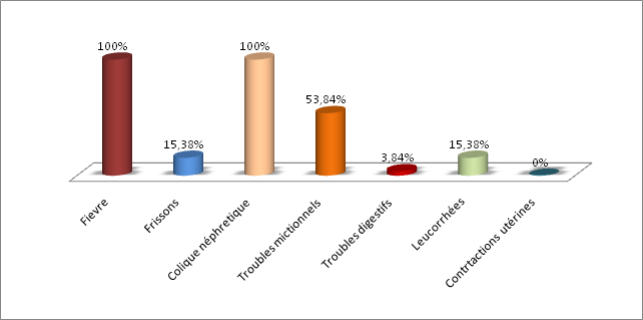
Signes cliniques de la PNAg dans notre série

**Figure 2:**
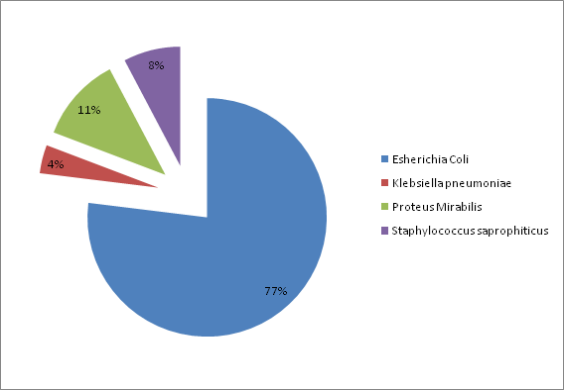
Répartition en fonction des germes en cause

Nous avons enregistré 26 patientes. La fréquence de la pyélonéphrite aigue gravidique par rapport aux pyélonéphrites aigues en général a été de 27,95% avec une prédominance chez les primipares de 53,84%. Son pic de fréquence se situe à 73,08% pour les gestantes âgées de 19 à 37 ans ainsi qu'au troisième trimestre (77%) de la grossesse. Huit patientes (33,77%) étaient diabétiques et cinq patientes (19,23%) avaient des antécédents de cystite. Les principaux signes cliniques (représentés dans la Figure 1) étaient dominés par la fièvre et les coliques néphrétiques 100%. Une fièvre entre 38,6° et 40° a été trouvée chez 24 patientes (92,30%). Une fièvre associée à des frissons a été retrouvé chez 3 patientes (11,54%). Les coliques néphrétiques ont été trouvées dans 100% des cas (80,76% localisées à droite; 15,40% à gauche et 3,84% des cas bilatérales). Les brulures mictionnelles ont été retrouvées dans 46,15% des cas, la pollakiurie dans 69,23% et la dysurie dans 7,70% des cas. L'examen physique a noté: Une sensibilité des fosses lombaires dans 24 cas (92,30% des cas), (80,76% à droite, 7,70% à gauche, 7,70% bilatérale) Dans 88,46% des cas, les urines des patientes ont été troubles. L'ECBU a confirmé l'infection urinaire et orienté le traitement antibiotique. La leucocyturie (leucocytes > 10.000/ml) a été observée dans 100% des cas de notre série. Le germe le plus fréquemment retrouvé est Escherichia Coli avec un pourcentage de 76,92%, suivi par le Proteus mirabilis avec 11% (Figure 2). Une culture réalisée chez une patiente (qui était déjà sous antibiotiques) est revenue négative. Elle a été positive chez une patiente et a permis d'isoler un germe identique à celui retrouvé lors de l'examen cytobactériologique des urines, il s'agit d'un Escherichia coli. La NFS a été réalisé chez toutes patientes et a montré une hyperleucocytose chez 22 patientes (84,61% des cas), une anémie chez 11 patientes (42,30% des cas). L'anémie a été hypochrome microcytaire dans 23,07% et normochrome normocytaire dans 19,23% des cas. La fonction rénale a été réalisée chez toutes les patientes de notre série et a été légèrement perturbée chez deux patientes. La CRP a été réalisé chez toutes les patientes de notre série, et a été élevée dans 100% des cas. La glycémie a été réalisée chez toutes les patientes et elle a été élevée chez huit patientes connues diabétiques. L’échographie rénale a été réalisée chez toutes les patientes, soit 100% des cas et a montré une dilatation urétéro-pyélo-calicielle(DUPC) chez 20 de nos patientes avec une prépondérance de DUPC sans obstacle chez 57,7% comme le montre le Tableau 1. Dix-huit de nos patientes (69,23%) ont bénéficié d'emblée d'un traitement médical visant à la stérilisation des urines, le rétablissement d'une bonne diurèse et la prévention des risques fœtaux notamment l'accouchement prématuré. Chez 66,66% des patientes, l’évolution a été favorable d'emblée avec rémissions des signes cliniques. En l'absence d'amélioration clinique et biologique, 33,33% des patientes ont bénéficié d'une montée de sonde double J (Tableau 2). La durée moyenne du traitement antibiotique a été de 15 à 21 jours. La durée moyenne du séjour à l'hôpital a varié entre 3 et 9 jours, selon l’état initial de la patiente et l’évolution clinique. La durée moyenne de séjour, chez l'ensemble des patientes a été de 4,8 jours. Huit patientes ont bénéficié d'emblée d'un traitement urologique conduisant à la montée de la sonde double J pour les raisons suivantes: échec du traitement médical (1 cas), dilatation urétéro-pyélo-calicielle (1cas), lithiases rénales droites (5 cas), lithiase urétérale gauche (1 cas). L'anémie a été retrouvé chez 11 patientes (42,30% des cas) (hypochrome microcytaire dans 23,07% et normochromenormocytaire dans 19,23% des cas). Dans notre série, 2 cas de déshydratation aigue ont été noté. Le bilan biologique de ces deux patientes a montré une légère hyponatrémie et l’évolution a été favorable après un apport hydro-électrolytique par voie parentérale. Deux gestantes ont présenté une menace d'accouchement prématuré au service d'urologie dont l’évolution a été favorable sous tocolyse.

**Tableau 1:** Résultats de l’échographie

Résultats	Nombre de cas	Pourcentage
DUPC sans lithiase	15	57,7%
DUPC avec lithiase	5	19,23%
Lithiase sans DUPC	1	3,84%
Normale	5	19,23%

**Tableau 2:** Antibiothérapie initiale utilisée dans notre série

Antibiotiques	Nombre de cas	Pourcentage
Amoxicilline+Ac.clavulanique	2	7,70%
Amoxicilline+Aminosides	7	26,92%
Ceftriaxone	17	65,38%
Total	26	100%

## Discussion

Toutes les données de la littérature retrouvent une incidence de la PNAg qui se situe entre 1 à 2% de toutes les grossesses [1, 2]. Dans notre série, au cours de notre période d’étude, l'incidence de la PNAg est estimée à 0,27%. Ce taux est bas par rapport à celui de la littérature du fait d'un faible échantillonnage dans notre série. L’âge moyen de nos gestantes a été autour de 28 ans. Cet âge moyen a été largement supérieur à celui retrouvé dans les séries de P. Sharma en Australie en 2007 chez qui l’âge était de 22 ans et chez C.E Mc Gruber où l’âge moyen était de 21 ans. Toutes les séries de la littérature retrouvent une prédominance de PNA au cours de la grossesse chez les primipares avec un pourcentage estimé à 75% [3] dans la série de P. Sharma et al. Ce taux est légèrement supérieur à celui de notre série qui était estimée à 53,84% lui-même comparable à celui de Mc Gruber [4] estimé à 53,1%. La pyélonéphrite aigue survient au fur et à mesure que la grossesse évolue au cours du deuxième et troisième trimestre [5, 6]. Selon P. Sharma et Al, la pyélonéphrite au cours de la grossesse a été diagnostiquée dans 60,63% des cas au cours du deuxième trimestre et dans 31,91% des cas au troisième trimestre [3]. Dans notre série, le nombre de pyélonéphrite aigue a été augmenté au cours du 3^ème^ trimestre entre 25 et 37 semaines d'aménorrhées soit une fréquence de 46,15%. Cette augmentation de la PNA en fin d’âge gestationnel est due à une augmentation de la hauteur utérine responsable d'une compression de l'uretère. La pyélonéphrite aigue réalise un syndrome douloureux, aigu fébrile du flanc. Le début brutal, associe d'emblée trois symptômes: un syndrome infectieux, inauguré parfois par des frissons avec élévation thermique rapide à 39°-40°C; des signes urinaires: brulures mictionnelles, dysurie et pollakiurie, qui souvent précèdent de quelques jours à la survenue de la fièvre; une douleur lombaire brutale, intense, paroxystique sans position antalgique uni ou parfois bilatérale [7]. Des signes fonctionnels associés de type digestif peuvent exister (nausées, vomissements et troubles du transit voire un syndrome sub-occlusif). Selon F. Gary et AL. La fièvre a été retrouvée dans 96% des cas,la douleur lombaire dans 82% des cas et les troubles mictionnels dans 40% des cas [8]. Dans notre série, la fièvre et les coliques néphrétiques ont été observé dans 100% des cas, les troubles mictionnels dans 53,84% des cas et les troubles digestifs dans 3,84% des cas. Il apparait donc que la triade fièvre, douleur lombaire,troubles mictionnelsest fortement évocatrice dans le diagnostic d'une PNAg. A l'examen clinique, l'altération de l’état général est le plus souvent évidente. Il peut exister une oligurie avec des urines parfois troubles et malodorantes. Une hypotension artérielle, une tachycardie maternelle élevée,une respiration rapide et superficielle sont des signes de mauvais pronostic, et annoncent des complications [8]. Parfois des contractions utérines sont présentes pouvant induire des modifications cervicales [9]. Le toucher vaginal peut retrouver une douleur au point urétéral inférieur (dans le cul de sac antérolatéral [9]. Dans notre série une sensibilité des fosses lombaires a été retrouvé dans 92,30% des cas (80,76% à droite; 7,70% à gauche et 7,70% bilatérale) et les modifications du col ont été observé dans 3,84% des cas. Le diagnostic de certitude de la PNAg repose sur la positivité de l'ECBU [10]. Dans notre série, les urines des patientes ont été troubles dans 88,46% des cas et l'hématurie macroscopique n'a été retrouvée chez aucune patiente. En cas d'infection urinaire, une hématurie supérieure à 104 hématies/ml dans environ 3 0% des cas peut être associée à la leucocyturie. Selon P. Sharma la leucocyturie a été notée dans 96,70% des cas et l'hématurie dans 10% des cas [3]. Dans notre série la leucocyturie a été retrouvée dans 61,53% des cas et l'hématurie microscopique dans 23,07% des cas. L'E. Coli est le germe le plus étudié, en raison de sa prévalence, et des problèmes qu'il pose sur le plan thérapeutique. Toutes les séries ont retrouvé E. Coli comme germe le plus fréquent avec un pourcentage de 81% dans la série de P. Sharma [3], 77% dans celle de F. Gary [8] et de 76,92% dans notre série. Cependant notre série retrouve en deuxième lieu une prédominance de Proteus Mirabilis avec un pourcentage de 11%. Tandis que les autres auteurs retrouvent Klebsiellapneumoniae en deuxième position. La NFS peut être utile pour apprécier la gravité de l'infection en montrant soit une hyperleucocytose importante, soit au contraire une leucopénie [11]. Dans notre série l'hyperleucocytose a été notée dans 84,61% des cas. Les patientes ayant une CRP maximale de 15mg/dl ont eu une hospitalisation plus longue et nécessite une antibiothérapie intraveineuse [12]. La CRP peut être utile pour déterminer la progression de la maladie ou de l'efficacité du traitement [13] et donc peut être considérée comme facteur prédictif de drainage de la voie excrétrice en cas de sa non diminution lors du traitement médical. Dans notre série la CRP a été réalisée chez toutes les patientes, et a été élevée dans 1001477; des cas. D'après Clyne B et al, la normalisation de la CRP constitue un marqueur de l'efficacité thérapeutique [14].

Il ressort de notre étude que l'altération de la fonction rénale est un facteur prédictif dans le drainage de la voie excrétrice supérieure. Les hémocultures ne sont positives que dans 15 à 20% des cas de pyélonéphrite aigue non compliquée. Elles sont nécessaires en présence de signes de gravité [11]. Dans notre série elles ont été positives chez une patiente (3,85%) et elles ont permis d'isoler un E. Coli. Les patientes diabétiques représentent 2% de la série de J. Hill et Al. [15] et 30,77% des gestantes dans notre série. Dans la série P. Sharma, l’échographie a été réalisée dans 80,85% des cas, et elle a retrouvé une dilatation urétéro-pyélo-calicielle(DUPC) dans 69% des cas et une lithiase des voies urinaires dans 5,32% des cas [16]. Quant à notre série, l’échographie rénale a été réalisée chez toutes les patientes et elle a montré une dilatation urétéro-pyélo-calicielle dans 76,93% des cas et un obstacle lithiasique dans 3.84% des cas. Les autres examens sont optionnels: urographie intraveineuse (UIV), l'abdomen sans préparation(ASP), la tomodensitométrie rénale (TDM), l'imagerie par résonnance magnétique (IRM) n'ont pas été réalisé dans notre série, ils répondent à des conditions de réalisations strictes du fait des risques fœtaux. La durée moyenne du séjour à l'hôpital dans notre série a été de 4,8 jours comparable à celle de P. Sharma qui est de 5,23 jours [16] et de Mc Gruber de 5,7 jours Le repos au lit en décubitus latéral gauche. Toutes les patientes de notre série ont bénéficié des mesures hygiéno-diététiques: Le repos au lit en décubitus latéral gauche. L'antibiothérapie doit être démarrée dès la suspicion clinique et après prélèvement des urines pour ECBU. Selon J. Delotte. Malgré les modifications physiologiques au cours de la grossesse, il n'existe pas de données permettant de préconiser des posologies d'antibiotiques différentes de celles utilisées de la femme non enceinte [17]. Le traitement doit être rapidement mis en route, il est institué de façon systématique en reconnaissant le rôle prédominant d'E. Coli dans des infections plus adaptés aux résultats de l'antibiogramme. Les bétalactamines sont les antibiotiques les plus utilisés, ils diffusent bien au niveau des tissus et notamment au niveau de l’œuf et du liquide amniotique [17]. En raison de leur efficacité, de leurs bonnes propriétés pharmacologiques et d'un faible taux de résistance des entérobactéries: les céphalosporines de 3^ème^ génération représentent l'antibiothérapie de choix pour traiter la PNA de la femme enceinte en attendant les résultats de l'antibiogramme [3]. Les céphalosporines suscitent moins de résistance [18]. Les céphalosporines ont été utilisées dans 65,38% dans notre série et aucun germe ne leur a été résistant. Les quinolones sont actifs sur les entérobactéries, ils sont contre-indiqués pendant la grossesse. Ils n'ont été utilisés chez aucune patiente dans notre série. Après 48 à 72 heures d'apyrexie, un relais par voie orale peut être effectué. Il tiendra compte des données de l'antibiogramme. Les molécules disponibles sont: L'amoxicilline + acide clavulanique,les céphalosporines de 3^ème^générationet le SMX-TMP. Dans notre série les molécules utilisées dans l'antibiothérapie de relais par voie orale ont été l'amoxicilline +Acide clavulanique dans 57,69% des cas et les céphalosporines de 3^ème^ génération dans 42,31% du fait de la prépondérance des germes les plus souvent retrouvée notamment E. Coli et Proteus mirabilis. Selon les recommandations de l'AFSSAPS la durée du traitement des pyélonéphrites gravidiques est au moins de 14 jours [19]. Dans notre série la durée du traitement antibiotique a été de 15 à 22 jours. Le traitement médical de la colique néphrétiqueassocie des antispasmodiques (phloroglucinol), du paracétamol, ou des anti-inflammatoires non stéroïdiens (AINS). Durant la grossesse, il faut éviter si possible, la prescription d'AINS, en cas de nécessité absolue ce traitement n'est alors prescrit que pour quelques jours (5 jours au maximum) [20]. Les antipyrétiques sont nécessaires en cas de fièvre car celle-ci diminue le débit utéro-placentaire. Le traitement urologique peut être indiqué d'emblée: la DUPC manifeste sur obstacle lithiasique endoluminal ou extrinsèque,la symptomatologie hyperalgique persistante malgré un traitement médical bien conduit, sepsis ou sepsis sévère, l'altération de la fonction rénale, pyelonephrite aigue sur rein unique anatomique ou fonctionnel. Le traitement urologique peut être indiqué secondairement en cas échec du traitement médical lié à différents:facteurs cliniques (persistance ou aggravation de la lombalgie, de la fièvre, de la symptomatologie urinaire), facteurs biologiques (hyperleucocytose persistante, non normalisation de la CRP), Persistance de la fièvre malgré l'antibiothérapie adaptée, Non normalisation de la CRP. J. F Hermieu préfère éviter la sonde double J en début de grossesse et réservent son utilisation au-delà de la 22^ème^ semaine [21]. Dans notre série, la montée de sondes double J a été réalisée chez huit gestantes. Une alternative à la pose d'une sonde urétérale est la mise en place d'une néphrostomie percutanée. La dilatation des voies urinaires au cours de la grossesse facilite sa pose. Denstedt et Razvi privilégient cette attitude avant la 22^ème^semaine de grossesse [22]. La néphrostomie n'a été réalisé chez aucune patiente dans notre série. L'utéroscopie, la lithotripsie extracorporelle(LEC), la néphrolithotomie percutanée (NLPC) sont contre indiquées pour la plupart des sociétés savantes du fait de leur effets néfastes sur le ftus. Dans notre série aucune de ces techniques n'ont été réalisées. Grace aux améliorations des méthodes de traitement, le recours à la chirurgie pour traiter un calcul des voies urinaires demeure exceptionnel. Chez la femme enceinte, la pose d'une sonde double J ou d'une néphrostomie permet d'attendre le terme de la grossesse pour envisager un traitement par lithotripsie ou endoscopique du calcul. Dans la série de P. Sharma 5 cas de lithiase urinaire (soit 5,23% des cas) ont été noté, l’évolution a été favorable sous traitement antibiotique dans tous les cas et le recours à un traitement urologique n'a été nécessaire dans aucun cas [3].

## Conclusion

La triade fièvre, lombalgies, troubles mictionnels chez une femme enceinte est fortement évocatrice d'un diagnostic de PNAg et doit inciter la prise en charge en milieu hospitalier. Le traitement médical repose sur l'antibiothérapie probabiliste qui sera adaptée ultérieurement en fonction des résultats de l'antibiogramme. Les facteurs prédictifs d'un drainage de la VES sont: la persistance de la symptomatologie clinique, du syndrome infectieux et des anomalies visibles à l’échographie rénale ainsi que l'altération de la fonction rénale.
